# COPD in Firefighters: A Specific Event-Related Condition Rather than a Common Occupational Respiratory Disorder

**DOI:** 10.3390/medicina58020239

**Published:** 2022-02-05

**Authors:** Armand-Gabriel Rajnoveanu, Ruxandra-Mioara Rajnoveanu, Nicoleta Stefania Motoc, Paraschiva Postolache, Gabriel Gusetu, Milena Adina Man

**Affiliations:** 1 Occupational Medicine Department, Iuliu Hatieganu University of Medicine and Pharmacy, 400012 Cluj-Napoca, Romania; armand.rajnoveanu@umfcluj.ro; 2 Palliative Medicine Department, Iuliu Hatieganu University of Medicine and Pharmacy, 400012 Cluj-Napoca, Romania; 3 Pneumology Department, Iuliu Hatieganu University of Medicine and Pharmacy, 400012 Cluj-Napoca, Romania; motoc_nicoleta@yahoo.com (N.S.M.); manmilena50@yahoo.com (M.A.M.); 4 Medical Department, Gr T Popa University of Medicine and Pharmacy, 700115 Iasi, Romania; postpar04@yahoo.com; 5 Cardiology Department, Iuliu Hatieganu University of Medicine and Pharmacy, 400012 Cluj-Napoca, Romania; gusetu@gmail.com

**Keywords:** chronic obstructive pulmonary disease, health status, exacerbations, occupational exposure, firefighter, wildland fire smoke, World Trade Center, lung function, biomarkers, quality of life

## Abstract

Chronic obstructive pulmonary disease (COPD) is a leading cause of morbidity and mortality worldwide. Smoking remains the most important risk factor, but occupational exposures may play an essential role as well. Firefighters are among occupations regularly exposed to a variety of irritative inhalational products, and they may be expected to develop respiratory health problems because of such an occupational exposure. To better understand and characterize this relationship, we performed an extensive search of the scientific literature, and we identified two major research areas: firefighters exposed to wildland fire smoke and firefighters involved in the World Trade Centre disaster-related operations. Most of the studies did not report a significant increase in COPD diagnosis in firefighters. An accelerated rate of decline in lung function was seen, a short time after major exposure events. This is the reason for an increased rate of exacerbations observed in individuals already diagnosed with obstructive respiratory disorders. A limited number of studies not covering these specific circumstances of exposure were found. They reported long-term morbidity and mortality data, and the results are controversial. Major confounding factors for most of the studies were the “healthy worker effect” and the lack of useful data regarding smoking habits. Efforts should be made in the future to better characterize specific biomarkers for the progression of COPD; to establish exposure limits; and to implement preventive strategies like rotation of workers, smoking cessation programs, and long-term monitoring programs for respiratory disorders.

## 1. Introduction

Chronic obstructive pulmonary disease (COPD) is a major public health problem worldwide (the third leading cause of death globally) [1], and it is anticipated to increase in its consequences in the next years, especially in low- and middle-income countries [2]. Being a preventable and treatable condition, it is important to identify significant risk factors for COPD and to diagnose affected individuals in early stages. Alongside smoking, a very well-known risk factor, occupational exposures seem to represent an important under-recognized risk factor. In a recent published meta-analysis, the occupational burden of COPD was reported as 14% PAF (population attributable fraction). This number is even higher when never-smokers were included in analysis (31%) [3].

Looking at the occupations at increased risk for COPD, we noticed jobs in emergency services being cited in the first 10 industry sectors [4], and among these jobs, firefighting is the one involving repeated exposure to irritative gases, fumes, vapors, and many other types of inhalational exposures, and therefore, the effects of all these exposures need to be considered [5]. Besides COPD, other respiratory conditions might be caused or at least related or aggravated by these exposures, like asthma, emphysema, chronic bronchitis, bronchiolitis, bronchiectasis, interstitial lung fibrosis, or even sarcoidosis. Different types of cancer remain a major concern that needs to be evaluated more in-depth.

The aim of our narrative review was to identify and select the most representative research studies addressing the relationship between COPD and firefighters and to find out how common this is among this occupation, in terms of morbidity and mortality, and its main characteristics.

## 2. Materials and Methods

We performed extensive research in two major databases using the key-terms: “chronic obstructive pulmonary disease” or “COPD” and “firefighters”, with no restrictions regarding the publication time, text availability, article type, and language. We identified 29 results in the PUBMED database and 56 items in EMBASE until 1 December 2021. After reviewing all the articles and eliminating duplicates and those that did not cover the topic, 43 articles were deeply analyzed and added to the review process. In addition, the references of the initial articles were also screened based on the relevance for this review.

We identified two major circumstances or exposures under which the research work was conducted: firefighters and wildland fire (WLF) smoke exposure and firefighters exposed during rescue operations on the World Trade Centre (WTC) disaster site. Beside these two sources, there was a limited number of articles covering this topic but not related to such specific events.

## 3. Wildland Fire Smoke

### 3.1. Major Exposure Constituents and the Mechanisms of Action

Smoke from wildland fires is a complex mixture with many constituents, especially in the form of particulate matter (PM) (these fires are responsible for 29% of all emissions of PM_2.5_ in the United States) [6], but also as gaseous compounds. Most of the carbon released is in the form of carbon dioxide (CO_2_) and smaller amounts of carbon monoxide (CO) and methane (CH_4_). Wood smoke, including wildfire-released smoke, may contain many of the toxic compounds found in cigarette smoke, and thus, similarities in health effects are expected. CO, polycyclic aromatic hydrocarbons (PAH), nitrogen oxides (NO_x_), dioxins, acrolein, benzene, formaldehyde, free radicals, and particulate matter are all found in WLF smoke [7].

Recently, an extensive report on the effects and mechanisms of action for wood smoke on the respiratory tract was published by Zeglinski et al. [8]. Wildfire smoke, through its similarities with cigarette smoke, in chronic exposures, may lead to the development and/or exacerbation of respiratory pathologies, including COPD. Mechanisms that may be involved are found to disrupt the epithelial barrier structure and function through the loss of cell–cell adhesion and impair the wound healing capacity of alveolar epithelial cells after injury [8]. The transition of epithelial cells to the fibroblast phenotype or the epithelial–mesenchymal transition in small airways has been proposed as a potential mechanism contributing to airway fibrosis in COPD [9].

### 3.2. Functional and Clinical Implications

In individuals with appropriate symptoms and significant exposure to noxious stimuli, spirometry is required to make the diagnosis, with a post-bronchodilator ratio of FEV_1_/FVC < 0.7 confirming a persistent airflow limitation and, thus, COPD [10].

Exposure to wildland fire smoke is associated with a decline in pulmonary function tests measured across a work-shift in firefighters. In the forced vital capacity (FVC) there were reported decreases of 65 mL in United States (US) firefighters [11] and 59 mL in French firefighters [12], but more suggestive for bronchoconstrictive conditions are declines in the forced expiratory volume in the first second (FEV_1_) (150 mL and 53 mL, respectively) and maximum mid-expiratory flow (FEF25-75) (497 mL/s and 53 L/min (883 mL/s)), noted by the same authors [11,12]. Data were collected on a single day, immediately before and immediately after exposure to WLF smoke [11] or immediately after exposure and 24 h after the end of exposure [12]. These types of observations were not seen in other research studies, like the one of Adetona et al. [13], or the decline was non-significant (30 mL for FEV_1_), as reported by Gaughan et al. [14]. A possible explanation for the susceptibility of individuals for accelerated rates of decline in FEV_1_ was suggested by Burgess et al. in a study on 379 firefighters [15]. They found an association between a more rapid decline in lung function and the genetic polymorphisms of interleukin-10 (IL-10), a cytokine which exerts suppressive effects on inflammation [15].

Referring to the pathogenesis of COPD, perhaps more relevant is to investigate if there is a cumulative, persistent effect on lung function associated with repeated wildland fire smoke exposure. Such reports are provided by studies looking at the cross-season declines observed in firefighters. Cross-seasonal observations are more suggestive for long-term chronic effects of firefighters’ exposure, compared to cross-shift assessments, which reflect closely acute effects. Liu et al. reported declines of 90 mL for FVC, 150 mL for FEV_1_, and 440 mL/s for FEF25-75 in California firefighters during one or two seasons [16]. Measurements were performed at the beginning of the fire season (May) and again at a time after the last fire-fighting activity (late September and October) [16]. These observations were consistent with those reported by Betchley et al. in another group of wildland fire firefighters (33 mL for FVC, 104 mL for FEV_1_, and 275 mL/s for FEF25-75) [11], with data being collected previously and as soon as possible after the fall fire season. Compared to other occupational groups, like construction workers or paper-pulp mill workers, firefighters had lower rates of decline for FEV_1_ (39.6 mL/year vs. 48.7 or 45.2) but higher rates when compared only with the never-smokers subgroup (34.3 mL/year) [17].

Most of these decline rates in FEV_1_ were higher or close to the expected age-related decline reported by Hnizdo et al. [17] of 90 mL/year. It is of great importance to mention that an observation of 8 to 11 years is considered to have sufficient reliability in detecting a linear relationship [17].

A critical review of the health impact of wildland fire smoke exposure on the general population revealed that mainly individuals with pre-existing COPD are affected, with the exacerbation rate being high in such circumstances [17]. This was suggested by increased rates of hospitalization and visits to emergency departments (ED) and to physicians in relation to wildfire smoke exposure [18]. These data seen in the general population may be easily extrapolated to firefighters, with the level of exposure in their case being higher compared to residential populations.

A particular point of interest these days is climate change because it is at the origin of an increased length of the wildfire season in many geographical areas, and this trend is expected to continue in the next years [19]. Among workers occupationally exposed to wildland fire smoke, firefighters are highly affected. Intense physical effort and extreme heat exposure induce an increased respiratory rate in firefighters and, through that, an increased inhalation of smoke [20]. Another factor that may contribute to increased susceptibility of firefighters to inhalational exposures associated to WLF is the lack of personal protective respirators. These are unsuitable for such conditions, and consequently, they are rarely used [21]. Another particularity shared by most of the firefighters’ communities is the “healthy worker effect” [22] due to a careful pre-employment selection of individuals with a very good health status and due to leaving of such a job once respiratory problems develop.

The most relevant research studies focusing on the respiratory effects of WLF smoke exposure in firefighters are presented in Table 1.

## 4. World Trade Centre Disaster

### 4.1. Assesment of Exposure

The event of the World Trade Centre towers’ collapse (2001, 9/11) generated an immense cloud of dust and fumes, which persisted, in different forms, until December 2001. The composition of this cloud was very complex, mainly consisting of calcium carbonate, originating in the gypsum and cement that was used extensively as construction materials, but also in silicates and crystalline silica, sulphates, fibrous glass, plastics, and chrysotile asbestos [23]. As cement is highly alkaline, a characteristic of the WTC dust was the high pH that was generated at contact with respiratory mucosa [24]. Retrospective assessment of exposure to particulate matter relied on measurements of the nearby regional monitoring stations but was limited to PM with an aerodynamical diameter ≤ 10 μm (PM_10_) and ≤ 2.5 μm (PM_2.5_), lacking measurements of ultrafine particles (PM < 0.1 μm) that may have posed a significant effect on respiratory function [25]. In fumes released from fires at the disaster site, chemicals were identified like polycyclic aromatic hydrocarbons (PAHs), benzene, dioxins, naphthalene, and polychlorinated biphenyls (PCBs) [26].

Due to intense exposure in the first hours after the collapse, emergency workers (including New York City firefighters) were divided into three subgroups in many of the following research cohorts: those with high exposure (arriving at the site in the morning of the disaster), those with intermediate exposure (arrived in the afternoon of the day of event or the next day), and the group characterized by low exposure (they entered the site at least 48 h after the collapse) [27].

### 4.2. Reported Diagnosis and Functional Changes

A follow-up study 7–9 years after the WTC disaster examining self-reported physician diagnosis of respiratory conditions in 9715 firefighters revealed a total of 413 (4.3%) cases of COPD/emphysema [28]. The fraction was higher when the retired population was considered (391 cases, representing 8.6% of the study population). After adjusting for smoking status, age on 9/11, arrival group, and duration at the WTC site, retirees were 7.4 times (95% CI = 5.3–10.5) more likely to have COPD/emphysema than actives [27]. In the COPD subgroup, a correlation was found between the number of subjects having this diagnosis and the FEV_1_% of predicted values: 50.6% (209 cases) were in the 20–82 quintile, with this proportion decreasing as the FEV_1_ measured was higher (19.6% for 83–89, 13.3% for 90–96, 9.7% for 97–103, and 6.8% for the 104–147 quintile) [28]. A limitation of this study was the lack of a reference unexposed group to compare the reported prevalence rates for COPD. Although self-reported physician diagnosis (including diagnoses made by any physician) may be seen as a confounding factor in this study, it is worth saying that the agreement between self-reported non-asthma obstructive airway disease (OAD) diagnoses and the New York City Fire Department physicians’ diagnoses was relatively high (8.9% vs. 6.3%, respectively), as reported by Weakley et al. [29] in a population including 12,528 firefighters. An important remark is that pre 9/11, in the study population, there were no cases of COPD/emphysema and that post-9/11, there has been a dramatic reduction in smoking rates [30], probably associated with an increased frequency of respiratory symptoms.

Because the self-reporting of a diagnosis has a higher specificity compared to sensitivity and a higher negative predictive value (NPV) than positive predictive value (PPV), researchers recommend using this type of information in identifying trends in epidemiological studies for conditions with a high prevalence and to rule out such a diagnosis when the prevalence is low [29].

It is important to underscore that the firefighters that had a higher cumulative incidence for OAD (including COPD) compared to other emergency medical service (EMS) workers (24.7% vs. 9.5%, *p* value < 0.00001) also arrived soon at the scene of disaster [31]. This suggests an increased exposure in case of this occupation and a higher risk for developing obstructive airway disease.

### 4.3. Biomarkers and Phenotypes

A biomarker to identify subpopulations of ever-smoker firefighters susceptible to developing COPD was the elevated postexposure level of blood eosinophils. This was associated with an increased rate of FEV_1_ decline (1.46 mL/year decline per 100 eosinophils/μL), as reported by Zeig-Owens et al. [32]. A more interesting result related to the eosinophils blood level of ≥300 cells/μL was its association with the asthma/COPD overlap (hazard ratio 1.85; 95% CI, 1.16–2.95), as found by Singh et al. [33]. This association was not found for isolated asthma or isolated COPD [33]. This phenotype of patients with COPD and a high eosinophils blood level may require long-term inhaled corticosteroid treatment, and attention should be paid to the side-effects of these drugs, as they may impact respiratory muscles, and a myopathy risk may be associated with their use [34].

Another biomarker that may be of interest for the progression of a decline in lung function in firefighters might be the level of serum imunglobuline-A (IgA). A low serum level of IgA (≤70 mg/dL) was associated with a lower FEV_1_% predicted in the year following 9/11. An increased risk of FEV_1_/FVC of <0.70 was also positively correlated with a low serum level of IgA (HR = 3.8, 95% CI: 1.6–8.8) [35].

This population of WTC dust-exposed firefighters was also used to investigate cardiovascular biomarkers that could predict lung injury, with it being known that inflammation and remodeling are key factors, both for asthma and COPD. Weiden et al. [36] have shown that higher levels of apolipoprotein-AII, C-reactive protein, and macrophage inflammatory protein-4 are associated with increased relative risks (RRs) for lung injury, while resistance was predicted by higher levels of the soluble vascular cell adhesion molecule and lower levels of myeloperoxidase [36].

The most relevant research studies focusing on the respiratory effects of WTC dust exposure in firefighters are presented in Table 2.

## 5. COPD Morbidity and Mortality in Other Firefighter Populations

Beside these studies that addressed respiratory conditions in firefighters in specific circumstances like WTC collapse and WLF, there are several publications that have tried to retrospectively assess, on a long-term basis, the mortality in this occupation. In a cohort of 29,992 US career firefighters, Pinkerton et al. found an increase in COPD mortality with increasing fire-hours (an exposure surrogate calculated based on job exposure matrices models), but mortality from COPD in the studied population was not elevated compared to the general population [37]. These findings are probably explained by smoking habits, with lower rates in firefighters compared to general population [38], and the “healthy worker effect” described above [22].

We must mention that in a previous large cohort study on Canadian firefighters (3328 subjects), Guidotti et al. found an increase in COPD mortality (Standardized Mortality Ratio (SMR) = 157.95% CI: 79–281) but without attaining statistical significance [39]. In this study, a confounding factor might have been the incorporation of asthma in the definition of COPD in the analysis of these results. The author also suggested an important “healthy worker effect” in this study population, due to an early migration out of the occupation of workers who are experiencing breathing difficulties [39].

Firefighters’ morbidity by COPD was studied in a large Danish cohort (16,860 subjects) compared with another occupational reference group consisting of military employees (396,963 individuals) [40]. This reference group was selected to avoid the “healthy worker effect”, assuming that both groups share the same physical and socioeconomic characteristics. The risk of developing COPD was modestly increased but was statistically non-significant in full-time firefighters compared to the military group (SIR-standardized incidence ratio = 1.14). These findings were different compared to asthma data, for which the relative risk was significantly higher in full-time firefighters (SIR = 1.58) but not among part-time/volunteer firefighters without regarding the duration of employment [40]. It is worth noting that part-time firefighters and volunteers, for whom a less important occupational exposure might be assumed, showed lower risks compared to the reference group for both asthma and COPD [40]. This may suggest an involvement of occupational exposures for both conditions and, potentially, a dose–effect relationship that was not investigated or reported in other studies. However, there are some limitations that have to be noted. No information regarding the smoking habit was available, the prevalence of COPD relied only on hospitalized cases, and the age of study group was relatively young.

Schermer et al. investigated a cohort of 570 Australian firefighters for associations be-tween occupational exposure and health-related quality of life [41]. They reported a prevalence of doctor-diagnosed COPD/emphysema/chronic bronchitis of 7% in this cohort, with 91% of investigated subjects reporting relevant occupational exposures in the past year [41].

Low lung function and an excessive decline in FEV_1_ or FVC over a five-year follow-up, even in those with lung function within the normal limits, are important predictors of morbidity and mortality from COPD [17], and because of that, pulmonary function testing of the exposed population remains an essential tool for secondary or tertiary prevention in exposed groups. However, these assessments are successful in identifying individuals with an accelerated decline in function tests from about the fourth to fifth year of follow-up [17].

## 6. Firefighters and Quality of Life

Studies investigating the quality of life once the respiratory condition has occurred are scarce, with most of them focusing on the new onset of such a disease in firefighters. This is particularly important because it seems that quality of life in workers exposed to chemicals and dusts could be earlier affected compared to lung function parameters [42]. Schermer et al., who found a prevalence of COPD/emphysema/chronic bronchitis of 7%, also identified an association between the presence of such a diagnosis and a poorer physical health-related quality of life, especially in firefighters who did not protect themselves in an optimal manner from inhalational occupational exposures [41].

## 7. Final Remarks and Conclusions

In general, we may assume that it remains unclear if routine occupational exposure in firefighters is associated with an accelerated decline in lung function and COPD in the absence of a disastrous event like the WTC or WLF. This is more difficult to determine if we consider full-time firefighters and part-time, volunteer firefighters distinctively. This second group is characteristic of small communities, but in terms of individuals involved at a national level, they might be more important (370,000 (33%) career firefighters and 745,000 (67%) volunteer firefighters in U.S.) [43].

Genetic studies on factors involved in regulatory mechanisms for lung inflammation may help in identifying populations at risk for developing obstructive respiratory disorders. Other biomarkers of subclinical inflammation valuable in exacerbations of lung disorders, including COPD, like the neutrophil-to-lymphocyte ratio [44], may also be tested as early markers of inflammation in firefighters after a major event characterized by massive inhalational exposure.

Inhaled therapy may improve the ability of the performing physical activity in COPD patients [45], a beneficial effect which is very important for such a physically demanding occupation. Treatment compliance or adherence rates in firefighters must be investigated in future trials. This is an important outcome included in the core outcome set recommended by the ERS (European Respiratory Society) [46]. Compensation schemes, peer support, and individual economic status may also play a role in controlling the disease, and they have to be investigated in future research studies.

Another target to address in firefighters already diagnosed as suffering from COPD is to increase their adherence to inhaled treatments, as this is an important step forward in controlling all-cause mortality and hospital admissions for exacerbations [47].

Overwhelmingly, the study populations consisted of males and, generally, young individuals. These are demographic characteristics that must be accounted when comparisons are done with general population data.

Another possible confounding factor, especially for the WTC disaster study population, is better access to health services and follow-up support. This is related to the implementation of an extensive WTC Health Program funded by US Government designed to cover all health problems related to this unique event’s exposure. A constant preoccupation must be to establish occupational exposure limits specific to wildland fire smoke, as these circumstances tend to become more and more familiar. Mitigation strategies for such exposures were reviewed in a recent paper [48]. Respiratory protection may benefit from the use of particulate/organic vapor/formaldehyde filter masks. Respiratory symptoms were less common in this group compared to the ones of particulate only or particulate/organic vapor filter masks [48]. These observations were seen in a simulation lab, and it is of major concern how they may be extrapolated in real-life, with the firefighters’ resistance to using these kinds of devices being well-known.

A useful preventive strategy to reduce the respiratory health effects would be to rotate firefighters in and out of heavy smoke situations during interventions in a major event. Firefighters have to be trained properly regarding the hazards of heavy smoke exposures, and comprehensive medical surveillance programs, like those that followed the WTC disaster, have to be developed in cases of a particularly high exposure event. Monitoring programs requiring a long-term observation of the exposed population have to be a priority.

## Figures and Tables

**Table 1:** Studies addressing COPD diagnoses associated to wildland fire smoke exposure in firefighters.

Article	Article Type	Sample Size	Outcome
Burgess et al., 2004 [15]	Original research	1204	Lung function and gene polymorphism
Hnizdo, 2012 [17]	Original research	965	Effectiveness of long-term spirometry monitoring programs
Reid et al., 2016 [18]	Review	-	Respiratory morbidity and mortality
Adetona et al., 2016 [9]	Review	-	Health effects (including COPD) of WLF smoke
Betchley et al., 1997 [11]	Original research	76	Cross-shift and cross-season respiratory effects
Jacquin et al., 2011 [12]	Original research	108	Short-term respiratory effects in smokers vs. non-smokers
Adetona et al., 2011 [13]	Original research	24	Cumulative exposure effects on lung function
Gaughan et al., 2008 [14]	Original research	58	Acute respiratory effects
Liu et al., 1992 [16]	Original research	63	Smoke effect on FEV_1_ and airway responsiveness

**Table 2:** Studies addressing COPD diagnoses associated to World Trade Centre Disaster in firefighters.

Article	Article Type	Sample Size	Outcome
Weakley et al., 2013 [29]	Original research	12,528	Agreement between self-reported and medical records diagnoses
Webber et al., 2011 [28]	Original research	14,314	Physician-diagnosed respiratory conditions
Weiden et al., 2013 [36]	Original research	1720	Biomarkers for lung injury
Yip et al., 2016 [31]	Original research	2281	12-year post-exposure cumulative incidence of different health problems
Zeig-Owens et al., 2018 [32]	Original research	9434	Long-term post-exposure decline in FEV_1_
Singh et al., 2018 [33]	Original research	2137	Predictors for asthma/COPD overlap
Putman et al., 2019 [35]	Original research	917	Serum IgA level and airway injury

## Data Availability

Not applicable.
